# On vibration suppression of a tendon-driven soft robotic neck for the social robot HARU

**DOI:** 10.3389/frobt.2025.1698343

**Published:** 2026-01-22

**Authors:** Seshagopalan Thorapalli Muralidharan, Randy Gomez, Georgios Andrikopoulos

**Affiliations:** 1 Robot Design Lab, Mechatronics Unit, KTH Royal Institute of Technology, Stockholm, Sweden; 2 Honda Research Institute of Japan Co., Ltd., Wako, Japan

**Keywords:** soft robotics, tendon-driven actuators, continuum robots, vibration suppression, underactuated systems, social robotics, feedback control

## Abstract

Tendon-driven continuum actuators (TDCAs) provide compliant and lifelike motion that is well suited for human–robot interaction, but their structural compliance and underactuation make them susceptible to undesired vibrations, particularly along unactuated axes under load. This work addresses vibration suppression in such systems by proposing a real-time control strategy for a two-degree-of-freedom TDCA-based soft robotic neck used in the HARU social robot, where yaw motion is unactuated and prone to oscillations due to eccentric loading. The proposed approach combines a current-based tendon pretensioning routine, baseline PID control of the actuated pitch and roll axes, and a novel Coupled Axis Indirect Vibration Suppression (CIVS) mechanism. CIVS exploits mechanical cross-axis coupling by using high-pass filtered yaw acceleration from an inertial sensor to generate transient tension modulations in the actuated tendons, thereby increasing effective damping of the unactuated yaw mode without introducing additional hardware or compromising compliance. A classical sliding mode control is also implemented as a nonlinear benchmark under identical hardware constraints. Experimental validation on the HARU neck under representative loading conditions demonstrates that the proposed method achieves substantial vibration attenuation. Compared to the baseline controller, CIVS reduces yaw angular range by approximately 53% and yaw acceleration area by over 60%, while preserving smooth, expressive motion. The results further show that CIVS outperforms the sliding mode controller in suppressing vibrations on the unactuated axis. These findings indicate that indirect, feedback-driven tendon modulation provides an effective and low-complexity solution for mitigating load-induced vibrations in underactuated soft robotic systems, making the approach particularly suitable for interactive applications where safety, compliance, and motion expressivity are critical.

## 1 Introduction

Social robots are autonomous systems that are designed to interact with humans by conforming to their social behaviors. Over the last few decades, social robots have gained traction as a solution to address personnel shortfalls in sectors such as healthcare, customer service, and education. In order to, seamlessly integrate into human environments, social robots must not only perceive social cues but also safely express emotions in a recognizable manner. To achieve this, social robots must be designed and developed to combine mechanical safety, stability, and expressiveness to cater to the complex demands of real-world social interaction. Achieving robust, socially appropriate behavior remains a challenge and central focus of ongoing research Breazeal et al. (2016). Consequently, any social robot intended for sustained child-robot interaction must incorporate intrinsically safe and compliant actuation.

**FIGURE 1:**
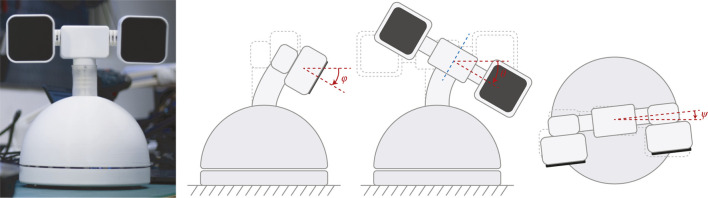
From left to right: The HARU prototype revision with the soft robotic neck, and its three degrees of freedom (DoFs): pitch ϕ (upwards and downwards movement), roll θ (sideways movement), yaw ψ (rotation about the head’s vertical axis). Adapted in part from Lindestam et al. (2024), © IEEE 2024, with permission.

The use of robots in educational settings for children has emerged as a viable strategy to mitigate teacher shortages and to deliver adaptive, engaging learning experiences in increasingly digital contexts Liu et al. (2022). Acknowledging the ethical, emotional, and developmental impacts of such technologies, the United Nations International Children’s Emergency Fund (UNICEF) in 2021 released a set of policy guidelines, describing the prerequisites for the application of AI and robotic technologies towards child centric applications (Virginia Dignum and et al., 2021). These emphasize psychological and physical safety, data transparency, and the importance of emotional expressiveness in robotic interactions. Following these objectives, the Honda HARU project was launched as a research platform to create a socially interactive educational companion robot for children Gomez et al. (2018). To achieve the goals of safe emotional expressiveness, the robot needs to provide fluid and lifelike movement while adhering to stringent constraints on mechanical stiffness, actuation force, ensuring safe, physically intuitiveness, and emotionally resonant human–robot interactions. Among the technological approaches that can deliver the required safety and expressiveness, soft-robotic actuation has emerged as a leading candidate. A rendering of HARU is shown in Figure 1.

Soft-robotic technologies, in particular, have gained prominence due to their potential for safer human machine interactions, reduced maintenance requirements, and for their adaptability to complex and unstructured environments Abidi and Cianchetti (2017). Their intrinsic flexibility and shock absorption are a result of soft materials and compliant structures that allow them to function effectively in diverse settings. Rus and Tolley (2015) define soft robots as autonomous systems composed of materials with Young’s moduli between $10^{4}$ and $10^{9}$ Pa, closely resembling soft biological tissues.

These robots utilize various actuation methods, including fluidic (pneumatic and hydraulic), thermal, magnetic, dielectric based, and tendon driven mechanisms Boyraz et al. (2018), Muralidharan et al. (2023), Wang et al. (2024). Despite the advances in actuation methods, the inherent structural compliance of soft robots makes them vulnerable to vibrations, particularly along their unactuated axes. Current control strategies inadequately address this issue, therefore, this is an active area of research.

Within soft robotics, fluid actuated soft robots represent a majority of soft robots used in academic, industrial and research settings Garriga-Casanovas et al. (2018). These however require bulky and noisy compressors rendering them unsuitable for silent, compact and mobile applications Malas et al. (2024). Tendon driven robots, by contrast, tend to be quieter, lighter and more energy efficient. Therefore, tendon-driven continuum actuators (TDCAs) are preferred for small, fast and mobile robotic platforms Muralidharan et al. (2021).

Like fluid actuated soft robots, tendon driven systems also inherit significant challenges. Their compliance and continuum nature leads to an increase in degrees of freedom and greater control complexity. Additionally, when subjected to sudden actuation or external impulse loads, these systems are prone to vibrations which manifest as unintended oscillatory dynamics and unstable motions. This issue is particularly problematic in larger and heavier tendon driven robots, where small base motions are amplified at the tip, and impulse loads introduce complex oscillatory dynamics. While traditional and state of the art control methods are effective for directly actuated axes and attainting the goal position, they often do not address vibrations occurring along unactuated or coupled axes. This paper addresses these limitations by proposing a new approach towards vibration suppression in tendon driven soft robots Tang et al. (2013).

HARU, a tabletop educational robot described in Gomez et al. (2018) and Andrikopoulos et al. (2024), deploys a tendon-driven continuum actuator (TDCA) in HARU’s neck allows for fluid, biomimetic motion capable of generating expressive and emotional behavior. The design uses a lightweight frame (<1 kg) for fast dynamic responses and quiet operation which is essential for Human Robot Interaction (HRI). The TDCA architecture provides compliance and lifelike movements, however motion control is challenging, especially along the unactuated degrees of freedom Rao et al. (2021), Tang et al. (2013). The pitch and roll axes are directly controlled by tendons, whereas the yaw axis lacks such control, making it susceptible to unregulated oscillations during dynamic movements. These axes of motion have been indicated in Figure 1.

The issue is further exacerbated by structural and mass asymmetries in the system. Specifically, the robot’s head includes a large tip mass that is eccentrically positioned relative to the neck’s central axis Oliver-Butler et al. (2019). This cantilevered configuration resembles an inverted pendulum with a flexible backbone subjected to off-axis loading, particularly affecting the unactuated yaw axis, which lacks direct tendon control. As a result, even minor base motions or external impulses can induce significant yaw-axis vibrations, which degrade the robot’s emotional expressivity.

Future iterations of HARU are expected to include actuated and movable components within the head assembly. While these enhancements aim to increase functionality, they may also introduce additional inertial effects and interaction torques, potentially amplifying yaw-axis vibrations. The current neck module functions as a 2-DoF TDCA under load, exhibiting unactuated and coupled-axis dynamics. Therefore, there is a clear need for an effective, low-complexity vibration suppression strategy that preserves compliance while maintaining the robot’s expressive motion capabilities.

**FIGURE 2:**
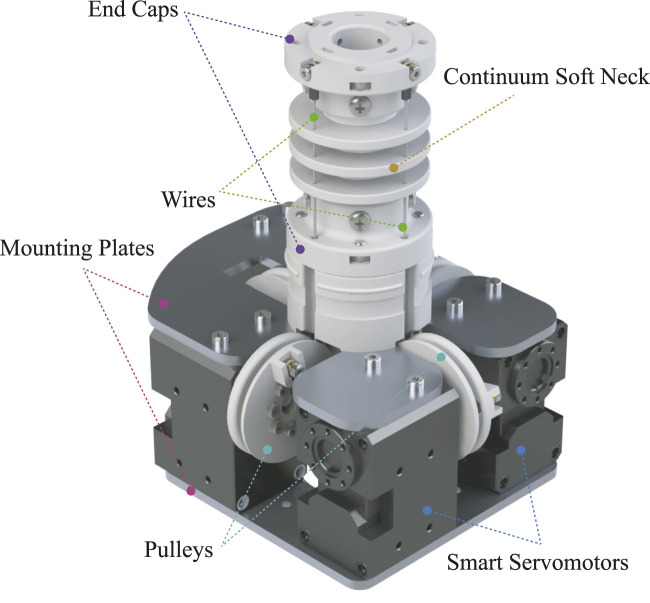
Graphical representation of the soft robotic neck module with annotated main properties. Adapted in part from Andrikopoulos et al. (2024), © IEEE 2024, with permission.

This study presents and experimentally validates a novel vibration control strategy for tendon-driven continuum actuator (TDCA) systems operating under load. The proposed method combines a current-based pretensioning routine, conventional PID control, dynamic tendon tensioning, and filtered inertial feedback to distinguish and attenuate unintentional vibrations without interfering with deliberate movements. Implementation and testing are carried out on HARU’s neck, whose module is graphically represented in Figure 2 and utilized as a base multi-DoF TDCA system. Empirical results demonstrate that this method can significantly suppress vibrations while maintaining control simplicity and compliance.

The proposed Coupled Axis Indirect Vibration Suppression (CIVS) strategy actively attenuates vibrations on unactuated axes by combining high pass filtered inertial feedback with adaptive PID-based modulation of tendon tensions along the directly actuated axes. In HARU’s two-degree-of-freedom (2 DoF) soft neck, this delivers indirect control of yaw-axis vibrations despite the absence of direct yaw actuation, thereby preserving both compliance and motion expressivity under dynamic loads. Unlike earlier vibration-control schemes, Khan and Li (2020) which target single-axis pneumatic systems and overlook close coupling, CIVS addresses the inter axis effects inherent to multi DoF tendon-driven continuum actuators through real time dynamic tendon tightening and coupled axis control. To the authors’ knowledge, this is the first real time method to suppress vibrations on an unactuated axis in a loaded multi-DoF TDCAs, achieving significant reductions in vibration amplitude without sacrificing fluidity or increasing control complexity, and this is useful for applications involving human interaction where safety and smoothness are essential.

The rest of this article is structured as follows. Section 2 provides a review of relevant literature, discusses the novelty of the proposed approach, and outlines the structure of the paper. Section 3 describes the methodology employed for the control strategy, providing information on the dynamic tendon tightening and the coupled axis vibration mitigation techniques. In Section 4 and Section 5, we present the experimental setup used to validate the proposed control method and the corresponding experimental results. Section 6 offers a detailed discussion of the findings, including performance comparisons across test cases and practical implications of the control strategy. Finally, Section 7 concludes the paper with a summary of the contributions, limitations, and suggestions for future work.

## 2 Related work

Despite significant advancements in the actuation and control of soft robotic joints, a majority of existing research has been directed toward position and force control, with relatively limited emphasis on dynamic vibration suppression. This bias is largely a result of two issues inherent with soft robotics. These are:

- Steady State Error: Due to the use of highly compliant materials prone to hysteresis and nonlinear elasticity, the final position of a soft robot is dependent on its loading, trajectory and internal state. This results in non-repeatable behavior.
- Slow Response Dynamics: Soft robots that employ actuation techniques other than tendon driven or pneumatic systems, such as thermal, electroactive polymers, or shape memory alloys typically exhibit low actuation bandwidths, making them poorly suited for rapid dynamic control or vibration mitigation.

Given these limitations, the focus of control in soft robotic joints has remained on achieving reliable static control, often at the expense of dynamic stability and vibration reduction.

Some work in the past have focused on reducing vibrations in soft and compliant robots, Gravagne et al. (2001) proposed a vibration-damping setpoint controller for continuum robots using boundary torques. While effective for backbone structures with directly actuated bending axes, these methods require detailed dynamic models and assume direct control authority on the vibrating DoF. Such assumptions do not hold for tendon-driven continuum actuator (TDCA) with unactuated lateral axes, where vibrations emerge due to load-dependent tendon coupling.

Ni et al. (2016) introduced a Coulomb-based soft damper to attenuate oscillations in bending actuators. Di Lallo et al. (2019) have demonstrated increased damping in silicone chambers by embedding viscous materials and granular media. These solutions improve structural damping but do not enable real time, directional suppression of dynamic vibrations, especially under asymmetric loading or multi-DoF tendon interactions.

Recent work has begun to address this gap. Khan and Li (2020) proposed a hybrid sliding mode control framework for active vibration suppression in soft robots. Their system integrates a nonlinear sliding mode control (SMC) with a PID based sliding surface, allowing the robot to remain on a desired trajectory despite vibratory disturbances. The hybrid nature of their controller offers robustness against model uncertainties and external perturbations, which are common in compliant structures.

Their research was validated through both simulation and experimentation on a multi chambered, planar pneumatic soft actuator, which operated in a two dimensional workspace. The experimental platform was unloaded and relatively low in mass, with dynamics dominated by internal pressure modulation rather than external or inertial loads. Their results demonstrated substantial reductions in residual oscillations during activation and deactivation phases. While this represents a promising step toward vibration aware control in soft robots, the scope of this approach was limited. The testbed system was constrained to planar motion and did not account for the complex, coupled dynamics inherent in multi degree of freedom (multi DoF) systems. Specifically, the system did not exhibit cross axis coupling, nor did it account for externally imposed loads, these are key factors that significantly influence vibratory behavior in real world applications.

Other recent works on soft robot control do not address the vibration-suppression problem considered in this article. Adaptive PAM control Mak et al. (2023) improves single-DoF tracking but does not target dynamic vibratory modes or multi-tendon coupling. Hysteresis modeling approaches Xie et al. (2024) focus on characterizing static and rate-dependent PAM hysteresis and do not consider cross-axis vibrations or unactuated modes. Data-driven modeling frameworks Chen et al. (2024) enhance prediction of soft robot behavior but do not provide real-time damping strategies and assume direct actuation on the controlled axis. None of these methods addresses real-time suppression of load-induced vibrations on an unactuated axis in a multi-DoF tendon-driven continuum joint, which is the core contribution of this work.

While previous works have demonstrated either passive or directly actuated vibration suppression mechanisms Khan and Li (2020), Ni et al. (2016), Gravagne et al. (2001), Di Lallo et al. (2019), they do not address situations in which undesired vibrations occur on an indirectly actuated axis, nor when vibratory modes depend strongly on external load conditions. Our proposed method achieves real-time vibration suppression in such cases by modulating the tension of orthogonal tendon pairs to counteract lateral-axis oscillations. To our knowledge, no prior study has demonstrated vibration suppression in a loaded, multi-DoF tendon-driven continuum joint where the axis experiencing vibration lacks direct actuation.

A comparision Table 1 listing the key differences in available literature and the relevance of this work has been shown:

**TABLE 1:** Comparison of prior work in soft joint control and vibration mitigation, extended with loading considerations.

Study	Actuator type	Control approach	Multi-DoF	Vibration	Load	Limitations
Mak et al. (2023)	PAM	Adaptive control	Single DoF	No	No	Only improves tracking; no vibration suppression
Xie et al. (2024)	PAM	Hysteresis modeling	Single DoF	No	No	Models hysteresis; not applicable to dynamic vibration
Chen et al. (2024)	Various	Data-driven methods	Varies	No	No	Modeling-focused; no vibration control
Khan and Li (2020)	Soft pneumatic	Sliding-mode damping	Yes	Yes	No	Requires direct actuation of vibrating DoF
Ni et al. (2016)	Soft bending actuator	Passive damper	Single DoF	Yes (passive)	No	Passive only; no real-time suppression
Gravagne et al. (2001)	Continuum robot	Setpoint vibration control	Yes	Yes	No	Assumes direct torque actuation; not tendon-driven
Di Lallo et al. (2019)	Soft continuum	Morpho-logical damping	Single chamber	Indirect	No	Improves damping but not axis-specific suppression
This work	TDCA	Real-time tendon modulation	Multi-DoF	Yes	Yes	Suppresses vibration on an *unactuated axis*; effective under external load

Although dedicated research on vibration control for multi DoF tendon-driven continuum actuators is still limited, useful analogies exist in domains where cross axis coupling must be controlled, namely, for overhead cranes, quadcopters and tailless (flying wing) aircraft. Overhead-crane controllers damp payload sway by predicting its motion and applying corrective trolley forces when direct actuation along every axis is unavailable Abdullahi et al. (2020). Quadcopter autopilots achieve smooth pitch, roll and yaw adjustments by modulating thrust on the other rotors Ramli et al. (2020), Gheorghiţă et al. (2015), while flying wing aircraft distribute control inputs across ailerons and elevons to counter the inherent yaw roll coupling that arises without a vertical tail Marqués (2013). In all three cases, vibration suppression is realized through coordinated, indirect control forces on coupled axes, a principle that is widely used in the strategy proposed here. These parallels provide insights into potential strategies for vibration control of soft robotic joints. In this context, where sensors and actuators are often distributed across the robot, a similar approach is needed to address vibrations in coupled systems. We draw inspiration from these allied areas and apply tension adjustments to the tendons based on feedback from the uncontrolled motion axis, which bears similarity to the counteracting forces used in crane systems or quadcopter rotors. By dynamically adjusting tendon tension to counter to vibrations detected in the yaw axis, this approach aims to reduce undesired oscillations, where direct control of the affected axis is not feasible.

## 3 Control structure

### 3.1 System overview and control goal

As first presented in Andrikopoulos et al. (2024) and shown in 2, the HARU’s soft robotic neck module is implemented as a tendon-driven continuum actuator (TDCA), in which four antagonistically paired steel wires are routed orthogonally around a flexible backbone and are connected to an equal number of smart servomotors. Active control is provided via the motors along the roll and pitch axes of the neck’s end effector, while the yaw degree of freedom remains unactuated due to structural design constraints Webster and Jones (2010), Burgner-Kahrs et al. (2015). This architecture enables compliant and expressive head motion but also makes the system susceptible to torsional oscillations particularly around the yaw axis due to the head’s eccentric mass and the lack of active yaw damping. Yaw refers to rotation about the vertical axis of the neck.

In the present HARU neck module, yaw motion is deliberately left mechanically unactuated so as to maintain structural simplicity, compactness, and compliance at the head. Adding a dedicated yaw actuator near the head would increase the tip inertia and could exacerbate undesired oscillations, especially under eccentric loading. The proposed CIVS strategy therefore utilizes cross-axis coupling in the tendon-driven architecture to indirectly suppress yaw vibrations without additional actuators.

Such oscillations can degrade both pose accuracy and the affective expressiveness required for high-quality human-robot interaction. To address this, we introduce Coupled Axis Indirect Vibration Suppression (CIVS), a control strategy that exploits mechanical cross-axis coupling within the TDCA to suppress undesired yaw vibrations. Prior to the execution of CIVS or any control behavior, the system undergoes a current-based pretensioning routine. This process incrementally retracts both tendons in each antagonistic pair to remove slack and establish a consistent preload baseline. Although mechanically simple, this step is essential for enabling predictable and symmetric control authority across the full operating range and is executed independently of the real-time feedback loops.

The core motion control of the system is performed using independent PID controllers for the roll and pitch axes. These controllers track reference trajectories in each axis and serve as the primary means of shaping motion and posture. On top of this baseline control, CIVS operates as an auxiliary vibration attenuation mechanism. It extracts high-frequency components of yaw motion using onboard inertial measurements and processes them through a shaping and filtering pipeline. The resulting corrective signals modulate tendon tensions in the roll and pitch axes to inject temporally localized viscoelastic damping attenuating yaw vibrations indirectly, without requiring actuation along the yaw axis.

### 3.2 Dynamic model

To conceptually illustrate how pitch/roll tensioning affects yaw behavior, the yaw axis is modeled as a second-order underdamped system:

$$J{\ddot{\theta }}_{y}+c{\dot{\theta }}_{y}+k\theta_{y}=\beta_{\text{indirect}}$$ (1)

Where:

-
$\theta_{y}$ is the yaw angle,
-
J is the moment of inertia of the head,
-
c is the damping coefficient,
-
k is the structural stiffness,
-
$\beta_{\text{indirect}}$ is the torque injected indirectly via pitch/roll tendon modulation.

This conceptual model helps explain the manner in which CIVS increases the effective damping ratio through tension dependent viscoelastic effects. Although small apparent shifts in dominant frequency were observed, these arise from the nonlinear viscoelastic response of the backbone rather than from deliberate stiffness increases in the control law. Although the proposed controller is model free, the conceptual yaw-axis model in (Equation 1) provides a physical basis for anticipating the manner in which pitch/roll tensioning can influence the yaw dynamics, particularly the natural frequency $\omega_{n}=\sqrt{k/J}$ and effective damping ratio ζ . Similar second-order approximations have been used previously for analyzing vibrations in continuum and tendon-driven systems, even when more complete models exist Rucker et al. (2010), Tang et al. (2013).

### 3.3 Current-based tendon pretentioning

Algorithm 1 implements an incremental, current-controlled pretensioning routine across each antagonistic motor pair. This hardware initialization steps runs only once at system start-up and its process begins by slightly retracting both tendons to remove slack. Then, within a closed feedback loop, the currents drawn by each motor are sampled and converted to physical units. To balance tendon tension, the algorithm incrementally advances the motor with lower current until the difference between the two falls within a specified tolerance. Once current symmetry is achieved, both motors are co-retracted in lockstep until their currents exceed a defined preload threshold. If either current drops below this threshold, the motors are stepped back and the loop exits. This ensures a safe, repeatable baseline pretension across all antagonistic pairs, establishing consistent initial conditions for downstream control experiments Oliver-Butler et al. (2019), Coad et al. (2019).

Algorithm 1 Current-based tendon pretightening algorithm.

**Require:** Set of motor indices M={1,2,…,N} where N=4

**Require:** Set of antagonistic motor pairs P={(i,j)∣i,j∈M}

**Require:** Current scaling factor α (provided by motor manufacturer)
**Require:** Current threshold γ , tolerance ϵ

1:  **for** each antagonistic motor pair (i,j)∈P **do**
2:    Read initial positions $p_{i}$ , $p_{j}$

3:    Slack removal: $p_{i}\leftarrow p_{i}-1$ , $p_{j}\leftarrow p_{j}-1$

4:    Write updated positions $p_{i}$ , $p_{j}$

5:    **while** true **do**
6:      Read raw currents $c_{i}^{\text{raw}}$ , $c_{j}^{\text{raw}}$

7:      Convert to amperes: $c_{i}\leftarrow \alpha \cdot c_{i}^{\text{raw}}$ , $c_{j}\leftarrow \alpha \cdot c_{j}^{\text{raw}}$

8:      **while** $|c_{i}-c_{j}|>\epsilon$ **do**
9:        **if** $c_{i}<c_{j}$ **then**
10: $p_{i}\leftarrow p_{i}+1$

11:        **else**
12: $p_{j}\leftarrow p_{j}+1$

13:        **end if**
14:        Write updated positions $p_{i}$ , $p_{j}$

15:        Read and update $c_{i}$ , $c_{j}$

16:      **end while**
17:      **if** $c_{i}\geq \gamma$ **and** $c_{j}\geq \gamma$ **then**
18:        Co-Retract: $p_{i}\leftarrow p_{i}-1$ , $p_{j}\leftarrow p_{j}-1$

19:        Write updated positions $p_{i}$ , $p_{j}$

20:      **else**
21:        **break**
22:      **end if**
23:    **end while**
24:    **Print:** “Tightening complete for pair (i,j) ”
25:  **end for**

### 3.4 Real-time control architecture

**FIGURE 3:**
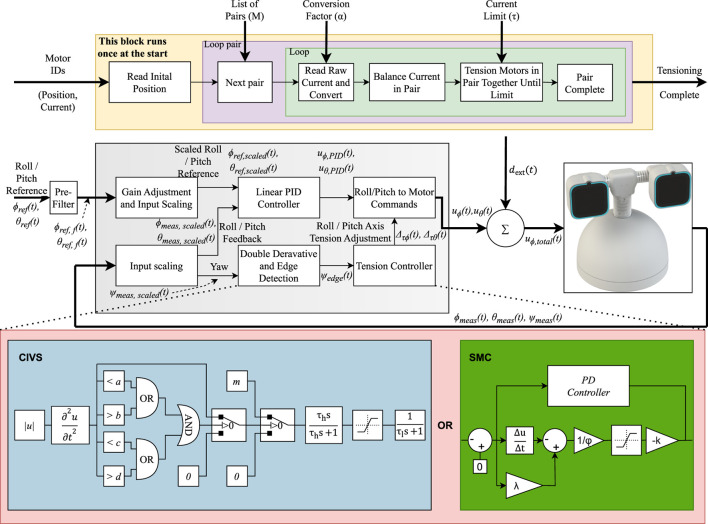
The top of the figure refers to the tensioning system used to achieve constant tension. The bottom part of the figure shows the proposed control architecture: The upper branch comprises classical PID loops in roll (ϕ) and pitch (θ) with gain-scaled inputs. The lower branch implements the CIVS mechanism, which processes high-frequency components of yaw motion (extracted from yaw acceleration $\ddot{\vartheta }$ ) into shaped tension commands applied via the pitch axis. These two pathways are combined to actuate the tendons driving the robot head’s roll and pitch axes. Next to the CIVS mechanisim is a SMC based vibration controller. This controller is used as a comparison to benchmark the CIVS controller.

The proposed control system employs a dual-branch architecture that separates the motion tracking of the head from vibration suppression. The upper branch consists of independent PID loops in roll (ϕ) and pitch (θ) , which ensure accurate tracking of commanded head motions. The lower branch implements a Coupled Axis Indirect Vibration Suppression (CIVS) mechanism, which monitors yaw motion and injects brief tension offsets into the tendon commands. Specifically, the CIVS branch converts high frequency yaw acceleration components, extracted from the IMU signal, into short, saturated and low pass smoothed tension offsets that are added to the pitch (and, in principle, roll) tendons. These offsets momentarily increase the effective tendon stiffness and viscoelastic damping, thereby attenuating yaw axis oscillations while minimally perturbing the desired roll pitch motion. Figure 3 provides an overview of this dual-branch architecture.

#### 3.4.1 Pre-filter

A pre-filter is used to generate a smoothed input signal for the system. This filtering minimizes abrupt changes in the command signal that could lead to undesirable motor transients. The pre-filter is implemented as a simple first-order low-pass filter.

The roll (ϕ) and pitch (θ) pitch references $\phi_{\text{ref}}\left(t\right)$ and $\theta_{\text{ref}}\left(t\right)$ are converted to $\phi_{\text{ref, f}}\left(t\right)$ and $\theta_{\text{ref, f}}\left(t\right)$ after the pre-filter. The cut off frequency of the filter is 10 rad/s in this case.

#### 3.4.2 Independent roll and pitch control

The roll (ϕ) and pitch (θ) axes are controlled independently using classical parallel-form PID controllers. Feedforward references $\phi_{\text{ref, f}}\left(t\right)$ and $\theta_{\text{ref, f}}\left(t\right)$ are generated externally and compared to sensor-based feedback signals $\phi_{\text{meas}}\left(t\right)$ and $\theta_{\text{meas}}\left(t\right)$ to compute axis-specific tracking errors:

$$e_{\phi }\left(t\right)=\phi_{\text{ref, f}}\left(t\right)-\phi_{\text{meas}}\left(t\right),\;e_{\theta }\left(t\right)=\theta_{\text{ref, f}}\left(t\right)-\theta_{\text{meas}}\left(t\right).$$

The corresponding PID control inputs are.

$$u_{\phi }\left(t\right)=K_{p,\phi }\;e_{\phi }\left(t\right)+K_{i,\phi }\int_{0}^{t}e_{\phi }\left(\tau \right)\;d\tau +K_{d,\phi }\;\frac{d}{dt}e_{\phi }\left(t\right),$$

$$u_{\theta }\left(t\right)=K_{p,\theta }\;e_{\theta }\left(t\right)+K_{i,\theta }\int_{0}^{t}e_{\theta }\left(\tau \right)\;d\tau +K_{d,\theta }\;\frac{d}{dt}e_{\theta }\left(t\right),$$

which are then converted into motor position commands for the tendon actuators.

The mapping from the axis-level control inputs to the tendon motor position commands is linear and can be written as

$$\left[\begin{array}{c} u_{\phi ,PID}\left(t\right)\\ u_{\theta ,PID}\left(t\right) \end{array}\right]=\left[\begin{array}{cc} c_{\phi } & 0\\ 0 & c_{\theta } \end{array}\right]\left[\begin{array}{c} u_{\phi }\left(t\right)\\ u_{\theta }\left(t\right) \end{array}\right],$$

where $c_{\phi }$ and $c_{\theta }$ are constant scaling factors determined by the motor horn (pulley) radius, tendon transmission geometry, and the motor’s position command conversion. The diagonal structure reflects that each axis uses an independent transmission ratio. The numerical values of $c_{\phi }$ and $c_{\theta }$ are implementation specific and therefore omitted here.

For numerical conditioning, we use scaled signals denoted by $\phi_{\text{ref,scaled}}\left(t\right)$ , $\theta_{\text{ref,scaled}}\left(t\right)$ , $\phi_{\text{meas,scaled}}\left(t\right)$ , and $\theta_{\text{meas,scaled}}\left(t\right)$ , obtained via the gain-scaling/normalization blocks shown in Figure 3.

#### 3.4.3 Coupled axis indirect vibration suppression (CIVS)

The lower control branch implements the CIVS mechanism, this modulates tendon tensions in response to detected yaw vibrations. The yaw signal ψ(t) is acquired from the onboard IMU, converted to a high-frequency yaw acceleration estimate, and then passed through a series of windowing, saturation, and filtering stages to isolate vibratory events. Conceptually, the CIVS branch converts these high-frequency yaw acceleration components into brief, bounded tension offsets that are added to the roll and pitch tendon commands. This transient increase in tendon tension momentarily raises the effective stiffness and viscoelastic damping (and may modestly influence apparent stiffness due to material viscoelasticity) of the neck backbone, which suppresses yaw-axis oscillations without significantly altering the underlying roll–pitch motion. Similar preprocessing pipelines have been applied in vibration suppression for flexible manipulators, for example, through adaptive input shaping Baklouti et al. (2021). The signal processing pipeline is as follows:

1. Input Scaling and Preprocessing: The measured yaw signal is normalized and converted to its absolute value:

$$\psi_{\text{abs}}\left(t\right)=|\psi_{\text{meas}}\left(t\right)|$$

We denote the normalized yaw input as $\psi_{\text{meas,scaled}}\left(t\right)$ , which feeds the subsequent differentiation and windowing stages.

2. Double Derivation (Acceleration Estimation): The second derivative is applied to emphasize abrupt changes:

$${\ddot{\psi }}_{\text{abs}}\left(t\right)=\frac{d^{2}}{dt^{2}}\;\psi_{\text{abs}}\left(t\right).$$

3. Window-Based Vibration Detection: A dual-sided window comparator $1_{\text{val}}\left(t\right)$ checks whether the acceleration lies within a selected band:

$$1_{\text{val}}\left(t\right)=\left\{\begin{array}{cc} 1,\; & b<{\ddot{\psi }}_{\text{abs}}\left(t\right)<a,\\ 1,\; & d<{\ddot{\psi }}_{\text{abs}}\left(t\right)<c,\\ 0,\; & \text{otherwise}. \end{array}\right.$$

with a>b>0 and c>d<0 .

4. Pulse Generation: A constant-magnitude signal u(t) is produced when vibration is detected:

$$u\left(t\right)=K_{u}\cdot 1_{\text{val}}\left(t\right).$$

We additionally form an edge-trigger signal $\psi_{\text{edge}}\left(t\right)$ from threshold crossings to gate pulse generation.

5. Edge Isolation via High-Pass Filter: To localize the control effort temporally, the signal u(t) is passed through a high-pass filter, creating a transient edge-focused control command.

$$\begin{array}{ll} \tau_{h}\dot{v}\left(t\right)+v\left(t\right) & =\tau_{h}\dot{u}\left(t\right),\\ \text{Laplace: }\;\frac{V\left(s\right)}{U\left(s\right)} & =\frac{\tau_{h}s}{\tau_{h}s+1}. \end{array}$$

6. Saturation and Low-Pass Smoothing: The resulting signal v(t) is capped to a safe amplitude and then smoothed:

$$w\left(t\right)=sat\left(v\left(t\right);0,K_{u}\right),$$

$$\tau_{l}\dot{y}\left(t\right)+y\left(t\right)=w\left(t\right),\;\text{with }\frac{Y\left(s\right)}{W\left(s\right)}=\frac{1}{\tau_{l}s+1}.$$

The final filtered signal y(t) , denoted as $a_{y}^{\text{HPF}}\left(t\right)$ , serves as the vibration estimate. It is added as a corrective offset to the pitch-axis tendon motor command:

$$u_{\theta }\left(t\right)=u_{\theta }^{\text{PID}}\left(t\right)+\tau_{\text{indirect}}\left(t\right),\;\text{where }\tau_{\text{indirect}}\left(t\right):=y\left(t\right).$$

#### 3.4.4 Indirect damping modulation via coupled axes

Due to the mechanical interdependence of the roll, pitch, and yaw axes in the tendon-driven cable assembly (TDCA), applying corrective tension on the roll and/or pitch tendons affects the dynamic stiffness and damping of the yaw mode. By modulating the pitch and roll tendon tension proportionally to the estimated vibration signal, the control system modifies the local strain field along the compliant backbone and increases the effective visco-elastic resistance to overcome the lateral twisting. This yields a higher equivalent damping ratio in the yaw mode while preserving the compliance and range of the commanded roll pitch motion.

#### 3.4.5 Combined control action

The outputs from the PID and CIVS branches are superimposed before being sent as motor position commands to the tendon actuators. Let the total control input to the motor interface for the pitch axis be:

$$u_{\theta }\left(t\right)=u_{\theta }^{\text{PID}}\left(t\right)+\beta_{\text{indirect}}\left(t\right),$$

and for the roll axis:

$$u_{\phi }\left(t\right)=u_{\phi }^{\text{PID}}\left(t\right)+\beta_{\text{indirect}}\left(t\right),$$

We denote the command after the summing junction by $u_{\phi ,total}\left(t\right)$ (analogously $u_{\theta ,total}\left(t\right)$ ).

as the indirect correction is only applied along the pitch tendon. These motor commands are passed to the tendon controller, which translates them into tension values for actuation.

The control signal $\beta_{\text{indirect}}\left(t\right)$ thus represents the vibration-suppressing component derived from yaw dynamics, indirectly modulating the pitch axis tension. By limiting the magnitude and duration of $\beta_{\text{indirect}}\left(t\right)$ through the shaping and filtering chain, the system injects damping at the most effective time while minimizing interference with voluntary head motion.

We refer to the induced tendon-tension increments as $\Delta \beta_{\phi }\left(t\right)$ and $\Delta \beta_{\theta }\left(t\right)$ in the current design the indirect correction is applied on the pitch axis only, i.e., $\Delta \beta_{\theta }\left(t\right)=\beta_{\text{indirect}}\left(t\right)$ and $\Delta \beta_{\phi }\left(t\right)=0\;d_{\text{ext}}\left(t\right)$ denotes exogenous disturbances (e.g., interaction torques and inertial effects) entering at the summing junction in Figure 3.

#### 3.4.6 Control sequence and design rationale

The processing sequence from derivative estimation to edge detection, followed by saturation and smoothing, is designed to isolate vibratory events and shape their response optimally. Any permutation of this order leads to degradation in effectiveness:

- Moving smoothing earlier erodes the signal’s temporal locality and delays the damping response.
- Saturation before high-pass filtering would produce variable pulse edges, dependent on signal history.
- Placing the absolute-value block later in the chain doubles the complexity of decision logic.

The implemented pipeline thus adheres to the minimal structure required to:

detect→pulse→edge→cap→smooth

which ensures early detection, bounded actuation, and stable filtering of high-frequency noise.

#### 3.4.7 Vibration damping effect on closed-loop dynamics

Linearizing the yaw dynamics around a dominant resonant frequency $\left(\omega_{n},\zeta \right)$ , the impact of the CIVS strategy can be modeled as a damping augmentation term $b_{eq}$ applied to the denominator of the yaw transfer function:

$$G_{\text{yaw,cl}}\left(s\right)=\frac{\omega_{n}^{2}}{s^{2}+2\left(\zeta +\Delta \zeta \right)\omega_{n}s+\omega_{n}^{2}+b_{eq}}$$

where.

$$\Delta \zeta =\frac{b_{eq}}{2\omega_{n}}$$

where $b_{eq}\propto K_{u}\alpha_{h}\alpha_{l}$ , with $\alpha_{h}$ and $\alpha_{l}$ being the effective gains of the high-pass and low-pass filters. This formulation shows that the CIVS contribution enters as an equivalent damping augmentation term $b_{eq}$ , increasing the effective damping ratio from ζ to ζ+Δζ , while leaving the natural frequency $\omega_{n}$ unchanged. Any small apparent stiffness shifts observed experimentally arise from tension dependent viscoelastic effects rather than from the CIVS control law itself.

#### 3.4.8 SMC formulation and implementation

A classical first-order SMC was implemented for comparison against the proposed PID and CIVS strategies. The controller operates on the tracking error between the measured angle and its desired reference, and forms a sliding variable by combining this error with its discrete-time derivative using a positive weighting factor that governs convergence speed. The control input consists of a linear stabilizing term and a nonlinear switching term, the latter shaped through a saturation function and a small boundary layer to avoid chattering. Together, these components generate a robust control action that compensates for disturbances and model uncertainties while driving the error dynamics toward the sliding surface. The resulting SMC output is mapped to the tendon-driven actuation in the same way as the PID commands, enabling a consistent comparison across control configurations while preserving compatibility with the tendon actuation hardware.

The SMC is implemented as follows:

$$\begin{array}{cc} s & =\dot{e}+\lambda e,\\ u & =-k_{1}e-k_{2}\;sat\;\left(\frac{s}{\phi }\right). \end{array}$$

The controller follows a standard first order sliding mode formulation, where the sliding surface is defined as $s=\dot{e}+\lambda e$ using the tracking error e and its derivative. The control input combines a linear stabilizing term and a nonlinear switching term according to $u=-k_{1}e-k_{2}\;sat\;\left(s/\phi \right)$ , where $k_{1}$ and $k_{2}$ are positive gains, ϕ sets the boundary layer thickness, and sat(⋅) is the saturation function used to reduce chattering. Since the system utilizes a model free approach. This controller was manually tuned to the best possible extent.

### 3.5 Vibration quantification metrics

To systematically evaluate the effectiveness of each control strategy, we define five metrics (Methods 1–5) derived from raw and filtered yaw data recorded by the onboard IMU. These metrics quantify yaw axis vibration in both time and frequency domains, enabling objective comparison across test cases. Comparable vibration quantification metrics such as RMS values, frequency domain analysis, and acceleration integrals have been widely used in robotic and mechatronic systems ISO (2016), Yavuz (2021). The following vibration quantification methods will be used in this work:

- Metric 1: Yaw Acceleration Area - The integral of the absolute value of the yaw acceleration over time. A lower area indicates reduced vibrational energy.
- Metric 2: Peak Count - The number of peaks detected in yaw position, velocity, and acceleration signals. Lower peak counts correspond to reduced oscillatory activity.
- Metric 3: Yaw RMS - Statistical root mean square (RMS) of the yaw position signal. High RMS indicates sustained deviation due to oscillation.
- Metric 4: FFT Based Frequency Analysis - The power spectral density (FFT) of the yaw signal is computed. The area under the FFT curve for frequencies below 10 Hz (indicating low frequency oscillatory energy) is identified.
- Metric 5: Yaw Range - The total angular displacement between the maximum and minimum yaw angles observed during a trial. Smaller ranges indicate better suppression of physical yaw oscillations.

## 4 System implementation and control test cases

**FIGURE 4:**
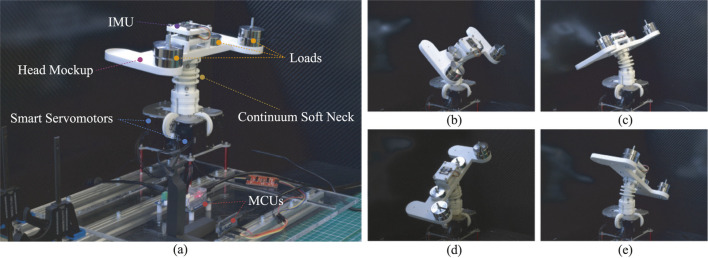
**(a)** Overview of the soft robotic neck setup with annotated main components, along with indicative photos displaying the neck while in **(b,c)** pitch and **(d,e)** roll motion.

The test rig developed for this study is shown in Figure 4a. The soft robotic neck follows the tendon-driven continuum structure introduced in the authors’ previous work Andrikopoulos et al. (2024). It consists of three spacer discs mounted between two end plates, actuated by two antagonistic pairs of steel tendons. These tendons are routed through symmetrically placed holes, clamped at the distal end, and driven by Dynamixel XM430-W350 servos, which convert motor rotation into bending moments along the backbone. The two principal motions achieved with this configuration pitch and roll are illustrated in Figures 4b–e.

To facilitate testing under variable loading conditions and to generalize the findings across different hardware configurations, a custom head mockup was designed and 3D-printed (Figure 4a). As the HARU robot’s head is undergoing continuous design changes, driven by evolving functional requirements, the placement of components and, consequently, the head’s mass distribution and center of mass (CoM) are subject to variation. The mockup allowed for controlled placement of variable loads at different locations, enabling repeatable evaluation of vibration control strategies under changing dynamic conditions.

To reflect the current head mass and distribution, a total equivalent load of 0.77 kg was mounted on the mockup, comprising 0.6 kg of added masses and an estimated 0.17 kg self-weight of the mounting structure (including weights of the mounting screws, nuts, IMU and IMU mount). The 0.6 kg of added mass was arranged as follows:

- Two 0.1 kg masses positioned longitudinally along the neck axis, at a distance of 50 mm on either side, representing the weight contribution of the eye movement mechanism.
- Two 0.2 kg masses positioned at a longitudinal distance of 100 mm and with a front-loaded offset of 40.7 mm, representing the weight contribution of the eyes.

This configuration approximates the present CoM location in the active head setup, with both longitudinal symmetry and the required forward offset maintained.

**FIGURE 5:**
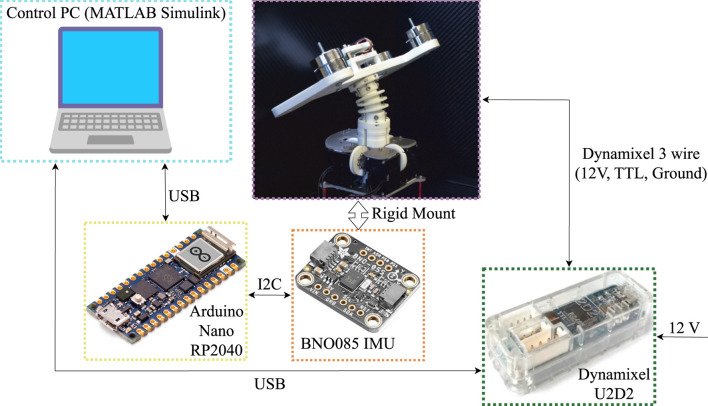
Main components used in the experimental setup utilized for evaluating the vibration suppression algorithms.

An onboard BNO085 IMU is mounted on the head mockup and is connected via I2C to an Arduino Nano RP2040 Connect microcontroller. The IMU provides 9-DoF quaternion-based orientation data at 50 Hz, which is transmitted over serial to a control PC running Simulink at the same frequency. A schematic overview of the data flow and setup is presented in Figure 5.

The BNO085 IMU uses it’s internal magnetometer, accelerometer and gyroscope to calculation a quaternion of the orientation of the IMU. This calculation is performed using an Extended Kalman Filter (EKF). This EKF is performed internally on the gyroscope. We compute yaw Ψ from the fused quaternion of the orientation.

### 4.1 Implementation details

All control software is executed in *Simulink* on a PC with fixed-time scheduling. The control loop operates at a sampling frequency of: $T_{s}=20\text{ ms},\;\left(f_{s}=50\text{ Hz}\right),$ resulting in a total sensor-to-actuator latency of less than 12 ms. Each experiment trial presented in the article was conducted for the same time window and was repeated five times to ensure statistical consistency. Unless otherwise stated, the plots and vibration metrics reported in Section 5 correspond to a representative mean trial, and the qualitative trends were consistent across all repetitions. Trial-to-trial variation for the key vibration metrics (yaw range and acceleration area) remained within approximately 15% of the mean, which is expected for soft components subject to visco-elastic effects and minor external disturbances.

Within this loop, yaw acceleration is estimated entirely in discrete time. At each sampling instant, the yaw angle ψ[t] is obtained from the IMU quaternion as described above, and finite-difference operators are used to compute $\dot{\psi }\left[t\right]$ and $\ddot{\psi }\left[t\right]$ , followed by the CIVS high-pass/low-pass filtering chain. The dominant vibration components observed in our experiments lie below 5 Hz (see Section 5), which is well within the Nyquist limit for the chosen sampling frequency $f_{s}=50\text{ Hz}$ .

#### 4.1.1 Actuation hardware

Tendon actuation is performed using Dynamixel XM430-W350 servos, which offer a position resolution of. $4096\text{ counts per revolution}\left(\Delta \theta_{\min}=0.088{}^{\circ}\right).$

#### 4.1.2 PID controller discretization

The PID controllers for roll and pitch are implemented in parallel form and discretized using the bilinear (Tustin) transform. This ensures consistency between continuous-time analysis and digital execution.

The continuous-time PID controller is given by:

$$u_{i}\left(t\right)=K_{p,i}\;e_{i}\left(t\right)+K_{i,i}\int_{0}^{t}e_{i}\left(\tau \right)\;d\tau +K_{d,i}\frac{d}{dt}e_{i}\left(t\right)$$

for i∈{ϕ,θ} .

and its Laplace-domain form is:

$$\frac{U_{i}\left(s\right)}{E_{i}\left(s\right)}=K_{p,i}+\frac{K_{i,i}}{s}+K_{d,i}s.$$

#### 4.1.3 CIVS parameterization

The Coupled Axis Indirect Vibration Suppression (CIVS) logic includes a tuned detection-and-filtering chain. The following parameters were selected for reliable detection and safe actuation:

These parameters define the shaping of the corrective signal $\tau_{\text{indirect}}\left(t\right)$ . The window comparator thresholds define the sensitivity of the vibration detection logic, while the pulse magnitude and filter characteristics ensure safe and temporally localized damping.

The chosen cut-off frequencies $f_{c,h}$ and $f_{c,l}$ are consistent with the measured vibration spectrum: they are tuned to target oscillations below approximately 5 Hz while attenuating higher-frequency noise introduced by discrete differentiation of ψ[k] . Combined with the 50 Hz sampling rate, this ensures that the estimated yaw acceleration used by CIVS is both dynamically relevant and numerically well conditioned.

#### 4.1.4 Controller deployment and timing robustness

All filters and transforms are executed in discrete time. The latency of the detection-to-actuation pipeline is kept within a single sampling frame, ensuring that damping inputs are delivered within tens of milliseconds after vibration onset.

### 4.2 Test case definitions

**FIGURE 6:**
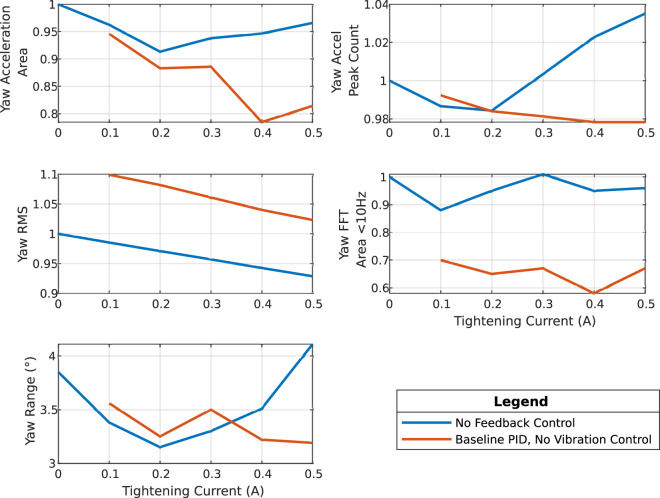
Comparative evaluation of yaw vibration suppression in the HARU soft robotic neck across increasing tendon tightening currents (0.0–0.5 A) under two control strategies: baseline PID (blue) and Coupled Axis Indirect Vibration Suppression (CIVS, red). Each subfigure presents a distinct performance metric, as defined in the methodology: Top row: yaw acceleration area and peak count; Second row: total actuator energy and raw yaw RMS; Third row: thresholded yaw RMS and FFT peak frequency; Bottom row: low-frequency FFT energy (<10 Hz) and total yaw range. Lower values generally indicate improved vibration damping and motion stability.

Before defining the test cases, we first performed a pretension sweep to determine a suitable operating point for the experiments. This sweep, shown in Figure 6, evaluated the effect of pretension current on the resulting vibration amplitude of the neck. The pretension was varied from 0.0 A (no pretension) to 0.5 A (maximum usable pretension) in increments of 0.1 A. These increments were chosen to exceed the effective controllable resolution of the actuation system (approximately 0.03 A), ensuring robust and repeatable settings.

The sweep showed a local minimum in vibration magnitude at 0.4 A. Based on this, all test cases involving pretension (Cases B–D) were executed at 0.4 A, while Case A represents the untensioned baseline. This procedure ensured that the four test cases reflect both practically realizable pretension levels and the optimal pretension setting identified experimentally.

Four control configurations were evaluated:

1. Test Case A: Baseline PID control without tendon tightening.
2. Test Case B: Fixed tendon pretension (0.4A) with basic PID for roll/pitch.
3. Test Case C: Fixed pretension + logical (on/off) control using filtered yaw acceleration.
4. Test Case D: Full CIVS: A high pass and low pass filter based dynamic tendon tensioning with filtered feedback.
5. Test Case E: Full SMC: Sliding Mode Control (SMC) based dynamic tendon tensioning.

## 5 Results

This section presents a comparative evaluation of the previously defined four vibration control strategies (Cases A–E) applied to the HARU neck’s yaw axis, while their fidelity were computed using five metrics (M1–M5). Results are reported as changes relative to the baseline (no control, 0.0A pretension) unless otherwise stated. Across all metrics, lower values denote better vibration suppression unless explicitly noted.

Figure 6 summarizes the results of the pretension sweep conducted prior to defining the four test cases. The figure reports how vibration metrics vary as a function of pretension current. As the pretension increases from 0.0 A to 0.5 A, vibration levels initially decrease, reaching a clear minimum at 0.4 A. Beyond this point, the vibration begins to increase again, and pretension values above 0.5 A were found to negatively affect actuator performance and motion expressivity. The observed minimum at 0.4 A motivated our choice of pretension for all pretension based test cases (B-D), ensuring that subsequent comparisons were made at the most favorable pretension identified experimentally.

Figure 6 illustrates the impact of tendon pretensioning on vibration suppression under different control strategies. Results are shown across a range of pretension currents (0.0–0.5 A) and evaluated using eight vibration-related metrics. The plotted curves compare two control configurations: a baseline PID controller for roll and pitch (blue), and the same PID controller combined with the proposed Coupled Axis Indirect Vibration Suppression (CIVS) mechanism (red).

The figure complements the summary in Table 2, which compares four key test cases: (A) no pretension or control, (B) 0.4 A pretension with baseline PID control, (C) 0.4 A pretension with PID and a logical yaw controller, (D) 0.4 A pretension with PID and full CIVS and (E) 0.4 A pretension with baseline PID and SMC vibration control. Across nearly all metrics ranging from yaw acceleration area and peak count to spectral and RMS-based vibration indicators Case D consistently outperforms the others, demonstrating the effectiveness of CIVS in suppressing yaw-axis oscillations without compromising compliance or control effort.

**TABLE 2:** Performance summary across metrics (Normalized for metrics 1 to 6, lower is better).

Metric	Case A	Case B	Case C	Case D	Case E
Yaw Acc. Area (M1)	1.00	1.06	0.62	0.38	0.95
Yaw Acc. Peak count (M2)	1.00	1.02	1.07	1.05	1.07
RMS yaw (M3)	1.00	1.00	1.00	1.00	1.00
FFT area <10 Hz (M4)	1.00	0.87	0.36	0.20	1.01
Yaw range (M5)	3.67°	5.49°	2.93°	1.7°	3.21°

**FIGURE 7:**
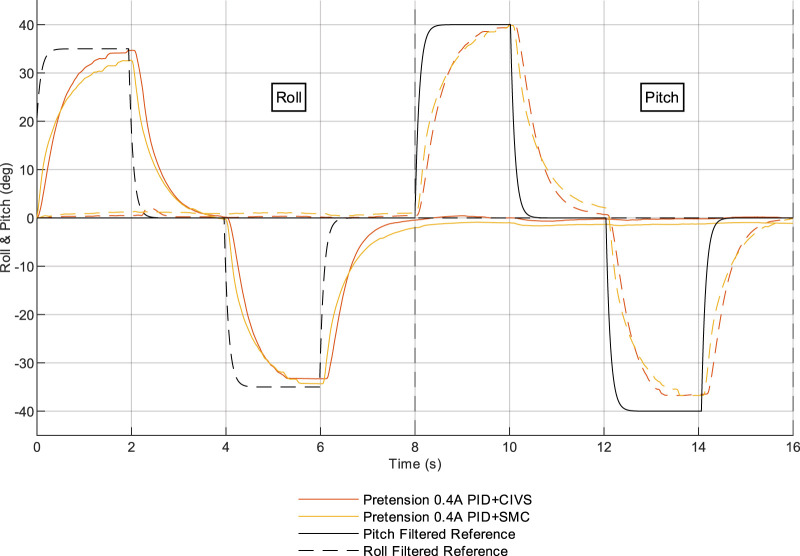
Roll and pitch angle trajectories measured from the IMU under two control conditions: fixed-pretension PID with CIVS (orange) and fixed-pretension PID with SMC (yellow). Dashed lines denote the filtered roll and pitch reference inputs. The measured IMU signals show that there is a tracking error in the reference due to inherent bandwidth limits of the tendon driven continuum neck and pretension induced stiffness.

Figure 7 shows the tracking accuracy of the roll and pitch signals of the PID + CIVS and PID + SMC system. In this case, the roll and pitch tracking is provide by the same PID controller. This controller is able to track the final value without sufficiently achieving the same time constant as expected by the input.

### 5.1 Metric 1 - Yaw acceleration area

This metric integrates the absolute yaw acceleration over time to quantify overall vibratory activity. As shown in Figure 6, high-pass-filtered PID control with CIVS (Case D) reduced this area by over 10% compared to the baseline (Case A). Its performance is similar to Case B and slightly worse than Case C. The performance of Case E (SMC) is similar to that of Case A (no pretension or control).

### 5.2 Metric 2 - Yaw acceleration peak count

The number of peaks in the yaw acceleration signal reflects both oscillation frequency and damping behavior. Figure 6 shows comparable results across all cases, with Case D performing slightly worse than the baseline. This trend however is seen in all cases.

### 5.3 Metric 3 - Yaw RMS

The root mean square (RMS) of yaw displacement, reflects deviation from the neutral pose. As illustrated in Figure 6 all cases appear to have similar performance as the baseline.

### 5.4 Metric 4 - frequency domain metrics

The FFT Area Under 10 Hz This captures low-frequency vibration energy. All cases involving tendon pretensioning (B, C, D) perform significantly better than the no-tension baseline, as seen in Figure 6. Case E however seems to vastly under perform with reference to the rest of the cases.

### 5.5 Metric 5 - Yaw range

The yaw range (maximum–minimum angular deviation) directly quantifies head sway. As shown in Figure 6, Case D reduces yaw range from 3.67° (baseline) to 1.7°, achieving a 53% reduction and outperforming all other configurations.

### 5.6 Yaw position response curves

To illustrate the qualitative effect of the CIVS method on the system’s vibrational response, we focus on the two extreme conditions: (A) the baseline with no pretension or control, and (D) the full CIVS configuration. These cases represent the lowest and highest levels of vibration suppression observed. The intermediate conditions (B: fixed pretension, and C: pretension with logical on/off control) are included in the quantitative analysis in Table 2 and in the pretension sweep shown in Figure 6, but are omitted here for clarity of visual comparison. Presenting the two extremes allows the contrast between no control and full CIVS control to be clearly seen in the measured yaw trajectories.

**FIGURE 8:**
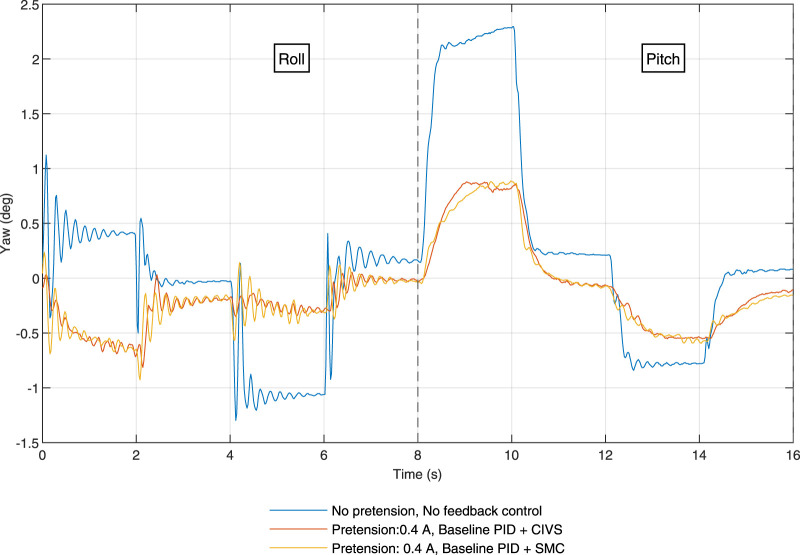
This figure shows the IMU-measured yaw response under three configurations: Test Case A (0 A pretensioning, no vibration control; blue), Test Case D (0.4 A pretensioning with CIVS; red) and Test Case E (0.4 A pretensioning with SMC vibration control; yellow). The CIVS configuration re-centers the yaw motion and reduces peak-to-peak amplitude from 3.67° to 1.7°, corresponding to an approximately 53% reduction in yaw-axis vibration. The roll and pitch portions of the input signal are shown.

Given the strong performance of Case D across key metrics, Figure 8 compares the yaw angle time response between Case A (baseline), Case D (CIVS with 0.4 A pretension) and Case E (SMC vibration control with 0.4 A pretension). The CIVS-controlled and SMC responses show a reduced peak amplitude and recenters around the origin, while the baseline trace exhibits offset oscillations and larger peak-to-peak motion. The observed phase shift and symmetry in the CIVS trace suggest increased yaw-axis viscoelastic behavior and improved dynamic stability arising from the transient tension changes. The SMC however, performs worse than CIVS as it has larger transients. These are largely caused due to quick changes in the sliding surface. While SMC provides good tracking accuracy locally, its inability to influence the unactuated axis shows that CIVS performs better when it comes to vibration supression.

CIVS is designed to attenuate, rather than completely eliminate, transient yaw oscillations so as to preserve the compliant, lifelike behavior of the soft neck. The remaining oscillations observed in Figure 7 fall within the allowable range of “expressive” flexibility needed for the target application, while still representing a substantial reduction in peak-to-peak yaw motion relative to the baseline.

## 6 Discussion

This section interprets the experimental results from four control configurations applied to the HARU robot’s unactuated yaw axis.

### 6.1 Performance comparison across control strategies

The most substantial reduction in yaw vibrations was observed in Case D, which employed high-pass filtered yaw feedback in conjunction with adaptive tendon tensioning via CIVS. As shown in Figure 6:

- Yaw acceleration area (Metric 1) was reduced by over 60% compared to baseline.
- Yaw angular range decreased from 3.67° to 1.7°, yielding an approximate 53% reduction.
- FFT analysis (Metric 4) showed a shift in dominant frequency from 4.8 Hz to 5.5 Hz and a decrease in low-frequency spectral content, indicating increased damping. The observed frequency shift is consistent with tension dependent viscoelastic effects but does not correspond to a deliberate stiffness augmentation term in the control law.
- CIVS also increases the effective damping c in (Equation 1), attenuating high-frequency oscillations without significantly altering compliance. This dual effect where increasing both k and c explains the simultaneous improvement in vibration suppression and preserving flexibility.

Although residual yaw oscillations remain present, they are an inherent feature of the compliant tendon backbone structure and not a limitation of the CIVS strategy. Importantly, CIVS does not rely on high pretension to achieve vibration attenuation. Instead, the improvement arises from transient, vibration triggered tension offsets that increase the effective damping of the yaw mode without altering the steady state stiffness or the expressive flexibility of the joint. The level of pretension used in Case D remains within the normal operating range of the actuator and is selected for safety and repeatability rather than as a requirement for achieving vibration suppression. Consequently, the reductions in yaw range ( ≈ 53%) and yaw acceleration area ( ≈ 60%) reflect the effect of CIVS rather than a dependence on elevated baseline tension.

Case D demonstrated improved vibration suppression across most metrics, including acceleration, RMS error, spectral distribution, and positional range. These results support the hypothesis that filtered, feedback-driven control improves damping performance in soft continuum systems, without compromising compliance.

By contrast, Case C featuring logical (on/off) control combined with fixed pretension showed inconsistent results and, in some trials, increased vibratory behavior. This may be attributed to *antagonistic interactions*, where logic-based corrective impulses conflicted with the continuous PID loop, resulting in irregular tension spikes. The effect is particularly visible in the total energy estimation, which increased despite apparent reductions in certain peak-based metrics.

Case E on the other hand used SMC. Although Sliding Mode Control (SMC) is widely regarded for its robustness in systems with model uncertainties, the experimental results in this work demonstrate that its advantages do not directly translate to tendon-driven continuum structures with unactuated DoFs. In the present setup, SMC exhibited larger transient responses and higher peak-to-peak yaw motion compared to CIVS, despite comparable tracking accuracy in the directly actuated roll and pitch axes. This behavior is primarily attributed to the fact that SMC can only influence the actuated subspace since yaw is mechanically unactuated, the switching action of the SMC surface cannot couple effectively into the torsional dynamics responsible for yaw vibrations. As a result, the controller reacts strongly to local tracking errors but remains insensitive to the global cross axis vibratory modes, leading to increased tension fluctuations and poorer damping performance. These findings show that robust controllers designed for fully actuated systems do not trivially generalize to continuum robots with underactuated and load dependent dynamics, this highlights the necessity of dedicated vibration-aware strategies such as CIVS.

The roll/pitch comparison figure shows that PID controller has a slightly low tracking bandwidth due to its soft-continuum morphology. The deviation from the reference is not a failure of the controllers but a structural and actuation-level constraint inherent to tendon-driven systems with limited motor torque dynamics. In particular, the motors cannot reproduce fast curvature changes because their internal position controllers and gearing are not designed for high-bandwidth torque modulation. As a result, any attempt to command sharp roll or pitch transitions is naturally filtered by the actuation chain, leading to the smooth, low-frequency trajectories observed experimentally.

### 6.2 Role of tendon pretension and controller architecture

A clear trend emerged regarding tendon pretension: increasing baseline tension from 0.0 A to 0.4 A improved vibration damping, but gains diminished beyond this level. Excessive pretension may introduce adverse coupling effects across the roll and pitch axes or reduce overall system compliance. This suggests the existence of an optimal pretension window sufficient to stabilize the yaw dynamics via cross-axis coupling, but low enough to preserve the natural flexibility of the TDCA structure.

Cases B and C, which employed fixed pretension and either baseline PID or logical control, were intended to explore whether simple passive or rule-based approaches could offer vibration mitigation. While some improvement was noted, performance gains were inconsistent. Logical clamping, though computationally efficient, lacks the adaptability of high-pass filtered feedback and may respond too coarsely to transient disturbances.

This smooth response is advantageous in the context of the intended application. The HARU neck module is designed for lifelike, expressive, and safe interaction rather than aggressive tracking of precise motion profiles. Thus, maintaining smooth and continuous deformation is a design priority.

### 6.3 Limitations and comparative discussion

From a control perspective, CIVS sits between purely passive vibration mitigation and fully model based robust control. Sliding mode or other model based damping approaches for soft actuators can offer strong disturbance rejection, but they typically require an explicit dynamic model and direct actuation along the controlled axis, and they may introduce chattering or increased implementation complexity. Our comparision using a model free Sliding Mode Control (SMC) shows that the SMC based controller performs poorly when compared to CIVS. This is largely due to the fact that the tuning of SMCs is not done through a model based method. Input-shaping methods, as used for flexible manipulators, assume approximately linear time-invariant dynamics and pre-planned motion profiles, assumptions that are difficult to guarantee in interactive social-robot scenarios. Morphological or passive damping strategies reduce vibrations by adding tuned compliance or viscoelastic elements, but they usually increase hardware complexity and can stiffen the structure permanently. In contrast, CIVS is implemented as a lightweight, model-free layer that reuses existing tendons and operates in real time on an unactuated, cross-coupled yaw mode under eccentric loading. To the authors’ knowledge, this combination of real-time, software-only damping of an unactuated DoF in a loaded multi-DoF TDCA has not been experimentally demonstrated before.

Empirically, we observed that CIVS tuning is not overly delicate. Moderate variations of the high-pass and low-pass cut-off frequencies and of the window thresholds in Table 3 (on the order of ±20% ) preserved closed loop stability and yielded qualitatively similar reductions in yaw range and acceleration area. Extreme parameter choices (e.g., very low cut-off frequencies or very large pulse magnitudes) can, as expected, slow down the response or increase energy consumption, but the overall damping trend remains robust.

**TABLE 3:** Nominal parameters for the CIVS chain.

Parameter	Symbol(s)	Value
List of motor pairs	M	(2, 4), (3, 5)
Current conversion factor	α	0.00269 A
Current limit	γ	0.4 A
Positive window upper threshold	a	1,500°/s^2^
Positive window lower threshold	b	200°/s^2^
Negative window upper threshold	c	−200°/s^2^
Negative window lower threshold	d	−1,500°/s^2^
Pulse magnitude	m	300 counts
High-pass filter cutoff frequency	$f_{c,h}$	0.008 Hz
High-pass time constant	$\tau_{h}=1/\left(2\pi f_{c,h}\right)$	19.89 s
Low-pass filter cutoff frequency	$f_{c,l}$	0.4 Hz
Low-pass time constant	$\tau_{l}=1/\left(2\pi f_{c,l}\right)$	0.398 s

It is important to note that both the proposed CIVS approach and the SMC benchmark were implemented in a fully model-free manner, consistent with the philosophy of this work. The SMC controller was manually tuned to the best achievable performance under these constraints. Even under careful hand-tuning, however, the controller consistently produced larger transients and poorer vibration rejection compared to CIVS.

This outcome is not primarily a tuning limitation but rather a structural one: because the yaw dynamics are unactuated, the switching action of the SMC surface cannot couple effectively into the torsional mode responsible for the dominant yaw oscillations. As a consequence, SMC reacts strongly to error in the actuated roll and pitch axes but remains fundamentally unable to inject damping into the unactuated yaw DoF. The controller therefore expends control effort in directions that do not influence the vibratory mode, leading to increased tension fluctuations and higher peak-to-peak yaw motion.

The roll and pitch tracking results also reveal an important limitation: the effective closed-loop bandwidth of the system is fundamentally constrained by the motor dynamics and continuum mechanics.

These findings highlight that, in underactuated tendon-driven continuum systems with load-dependent dynamics, classical robust controllers such as SMC do not automatically translate into effective vibration suppression. Instead, dedicated strategies that explicitly exploit cross-axis mechanical coupling such as CIVS are required to achieve consistent damping performance in a model-free setting.

### 6.4 Future work

While the proposed approach shows strong potential for vibration suppression in soft robots, several limitations highlight areas for further development. These are:

- Yaw control is achieved indirectly via cross-axis mechanical coupling, rather than direct actuation. While this design preserves mechanical simplicity and compliance, it may limit control precision in highly dynamic or asymmetric motion regimes.
- The system was validated primarily under quasi-static or moderately dynamic conditions. Future studies should evaluate performance under aggressive motion sequences, external perturbations, or unpredictable environments scenarios that are more representative of real-world operation.
- The current controller relies on manually tuned parameters for gains and filter cutoffs. While effective, this approach may not generalize well across robots or tasks. Integrating adaptive filtering or learning-based control policies could improve robustness and reduce manual effort.
- Although the method is model-free and hardware-agnostic, it may be sensitive to variations in mechanical properties (e.g., tendon elasticity or motor torque characteristics). Addressing this sensitivity will be important for scalability and long-term deployment.

Future work will focus on extending the CIVS framework to dynamic environments, incorporating adaptive gain tuning, and integrating low-drift inertial sensors for improved estimation. The CIVS concept itself may also generalize to other unactuated modes in continuum or soft robotic systems.

In summary, the results indicate that filtered, indirect control using high-pass yaw feedback and adaptive tendon modulation provides a low-complexity yet effective strategy for mitigating unwanted oscillations in unactuated soft robots. The approach strikes a balance between stability and compliance, offering a practical solution for enhancing motion fidelity in expressive robotic platforms.

## 7 Conclusion

This work presented a real-time control strategy for suppressing vibrations in the unactuated yaw degree of freedom (DoF) of tendon-driven continuum actuators (TDCAs). By leveraging filtered inertial feedback and modulating tension in the orthogonally actuated pitch and roll tendons, the method attenuated yaw-axis oscillations without requiring direct actuation or added mechanical complexity.

The proposed control framework, Coupled Axis Indirect Vibration Suppression (CIVS), combines high-pass filtered yaw acceleration with a PID-based tendon tensioning loop. This enables selective suppression of high-frequency yaw disturbances while preserving the compliant behavior intrinsic to soft robotic systems.

Experimental validation on the HARU robotic neck showed that CIVS achieved:

- A 53% reduction in yaw angular range (from 3.67° to 1.7°),
- Over 60% reduction in yaw acceleration area,
- A frequency shift in dominant yaw oscillation from 4.8 Hz to 5.56 Hz, indicating increased effective stiffness,
- Reduced low-frequency vibration energy and improved damping compared to baseline and logic-based control methods.

The comparison with a manually tuned, model free SMC controller further shows the structural constraints of underactuated tendon driven continuum robots. Despite its well known robustness in fully actuated systems, SMC was unable to attenuate yaw axis vibrations, as the unactuated torsional mode is challenging for the controller to fully suppress. Consequently, even with careful tuning, SMC produced larger transients and greater peak-to-peak yaw motion than CIVS. This result demonstrates that robustness alone is insufficient for vibration suppression when the dominant vibratory mode is unactuated, therefore, effective damping must instead exploit cross-axis mechanical coupling. CIVS achieves this through targeted tension modulation aligned with the TDCA’s intrinsic coupling, offering a practical and low-complexity solution for model-free vibration reduction.

This indirect control approach provides a practical solution for managing unwanted oscillations in unactuated DoFs particularly valuable in applications such as human–robot interaction, rehabilitation, and wearable robotics, where compliance is essential but unmitigated vibrations reduce performance and comfort. However, the system remains sensitive to sensor artifacts, including yaw drift caused by magnetic interference in the IMU, which can degrade feedback quality and increase unnecessary control effort. Future implementations may benefit from improved sensing pipelines, such as sensor fusion or magnetometer-free estimation techniques.

In addition, the control gains and filter parameters were manually tuned. While effective in this setting, automated or adaptive tuning potentially through learning-based methods could improve generalizability across platforms and tasks. Incorporating multimodal feedback (e.g., visual, inertial, proprioceptive) may further support context-aware control.

As soft robotic systems continue to advance into unstructured and interactive environments, the ability to manage unactuated DoFs without compromising compliance will be increasingly important. The CIVS strategy offers a scalable, low-complexity solution to this challenge and represents a step toward more robust, expressive, and stable control in soft robotics.

## Data Availability

The raw data supporting the conclusions of this article will be made available by the authors, without undue reservation.

## References

[B1] Abdullahi A. Mohamed Z. Selamat H. Pota H. Abidin M. Z. Fasih S. (2020). Efficient control of a 3d overhead crane with simultaneous payload hoisting and wind disturbance: design, simulation and experiment. Mech. Syst. Signal Processing 145, 106893. 10.1016/j.ymssp.2020.106893

[B2] Abidi H. Cianchetti M. (2017). On intrinsic safety of soft robots. Front. Robotics AI 4, 5. 10.3389/frobt.2017.00005

[B3] Andrikopoulos G. Hässler L. Gomez R. (2024). “On the design of a soft robotic neck for the social robot haru,” in *2024 IEEE international conference on advanced intelligent mechatronics (AIM)* (IEEE), 428–433.

[B4] Baklouti S. Courteille E. Lemoine P. Caro S. (2021). Input shaping for feed-forward control of cable-driven parallel robots. J. Dyn. Syst. Meas. Control 143, 021007. 10.1115/1.4048354

[B5] Boyraz P. Runge G. Raatz A. (2018). An overview of novel actuators for soft robotics. Actuators 7, 48. 10.3390/act7030048

[B6] Breazeal C. Dautenhahn K. Kanda T. (2016). Social robotics. Springer handbook of robotics, 1935–1972.

[B7] Burgner-Kahrs J. Rucker D. C. Choset H. (2015). Continuum robots for medical applications: a survey. IEEE Trans. Robotics 31, 1261–1280. 10.1109/TRO.2015.2489500

[B8] Chen Z. Renda F. Le Gall A. Mocellin L. Bernabei M. Dangel T. (2024). Data-driven methods applied to soft robot modeling and control: a review. IEEE Trans. Automation Sci. Eng. 22, 2241–2256. 10.1109/tase.2024.3377291

[B9] Coad M. M. Blumenschein L. H. Cutler S. Zepeda J. A. R. Naclerio N. D. El-Hussieny H. (2019). Vine robots. IEEE Robotics and Automation Mag. 27, 120–132. 10.1109/mra.2019.2947538

[B10] Di Lallo A. Catalano M. G. Garabini M. Grioli G. Gabiccini M. Bicchi A. (2019). Dynamic morphological computation through damping design of soft continuum robots. Front. Robotics AI 6, 23. 10.3389/frobt.2019.00023 33501039 PMC7806024

[B11] Garriga-Casanovas A. Collison I. Rodriguez y Baena F. (2018). Toward a common framework for the design of soft robotic manipulators with fluidic actuation. Soft Robotics 5, 622–649. 10.1089/soro.2017.0105 30161015 PMC6203266

[B12] Gheorghiţă D. Vîntu I. Mirea L. Brăescu C. (2015). “Quadcopter control system,” in 2015 19th international conference on system theory, control and computing (ICSTCC) (IEEE), 421–426.

[B13] Gomez R. Szapiro D. Galindo K. Nakamura K. (2018). “Haru: hardware design of an experimental tabletop robot assistant,” in Proceedings of the 2018 ACM/IEEE international conference on human-robot interaction (New York, NY, USA: Association for Computing Machinery), 233–240. 10.1145/3171221.3171288

[B14] Gravagne I. A. Rahn C. D. Walker I. D. (2001). “Good vibrations: a vibration damping setpoint controller for continuum robots,”Proc. 2001 ICRA. IEEE Int. Conf. Robotics Automation (Cat. No. 01CH37164) 4, 3877–3884. 10.1109/robot.2001.933222

[B15] ISO (2016). ISO 20816-1:2016: Mechanical vibration-measurement and evaluation of machine vibration-part 1: general guidelines. Geneva, Switzerland: International Organization for Standardization.

[B16] Khan A. H. Li S. (2020). Sliding mode control with pid sliding surface for active vibration damping of pneumatically actuated soft robots. IEEE Access 8, 88793–88800. 10.1109/access.2020.2992997

[B35] Lindestam A. Muralidharan S. T. Andrikopoulos G. Gomez R. (2024). “Model identification of a soft robotic eye actuator for safe social interactions,” in 2024 IEEE International Conference on Advanced Intelligent Mechatronics (AIM), 211–216. 10.1109/AIM55361.2024.10637028

[B17] Liu B. Tetteroo D. Markopoulos P. (2022). A systematic review of experimental work on persuasive social robots. Int. J. Soc. Robotics 14, 1339–1378. 10.1007/s12369-022-00870-5

[B18] Mak Y. X. Naghibi H. Lin Y. Abayazid M. (2023). Adaptive control of a soft pneumatic actuator using experimental characterization data. Front. Robotics AI 10, 1056118. 10.3389/frobt.2023.1056118 37008986 PMC10050439

[B19] Malas D. Wang S. Huang W. Lindenroth L. Xia W. Liu H. (2024). A novel pneudraulic actuation method to enhance soft robot control. Soft Robot. 12, 423–435. 10.1089/soro.2024.0094 39723930

[B20] Marqués P. (2013). Flight stability and control of tailless lambda unmanned aircraft. Int. J. Unmanned Syst. Eng. 1, 1–4. 10.14323/ijuseng.2013.5

[B21] Muralidharan S. T. Zhu R. Ji Q. Feng L. Wang X. V. Wang L. (2021). “A soft quadruped robot enabled by continuum actuators,” in 2021 IEEE 17th international conference on automation science and engineering (CASE) (IEEE), 834–840.

[B22] Muralidharan S. T. Andrikopoulos G. Feng L. (2023). “A survey on the current trends and applications of design optimization for compliant and soft robotics,” in *2023 IEEE/ASME international conference on advanced intelligent mechatronics (AIM)* (IEEE), 47–53.

[B23] Ni F. Henning A. Tang K. Cai L. (2016). “Soft damper for quick stabilization of soft robotic actuator,” in 2016 IEEE international conference on Real-time computing and robotics (RCAR) (IEEE), 466–471.

[B24] Oliver-Butler K. Till J. Rucker C. (2019). Continuum robot stiffness under external loads and prescribed tendon displacements. IEEE Transactions Robotics 35, 403–419. 10.1109/tro.2018.2885923

[B25] Ramli L. Mohamed Z. Efe M. Lazim I. M. Jaafar H. (2020). Efficient swing control of an overhead crane with simultaneous payload hoisting and external disturbances. Mech. Systems Signal Processing 135, 106326. 10.1016/j.ymssp.2019.106326

[B26] Rao P. Peyron Q. Lilge S. Burgner-Kahrs J. (2021). How to model tendon-driven continuum robots and benchmark modelling performance. Front. Robotics AI 7, 630245. 10.3389/frobt.2020.630245 33604355 PMC7885639

[B27] Rucker D. C. Jones B. A. Webster III R. J. (2010). A geometrically exact model for externally loaded concentric-tube continuum robots. IEEE Transactions Robotics 26, 769–780. 10.1109/TRO.2010.2062570 21566688 PMC3091283

[B28] Rus D. Tolley M. T. (2015). Design, fabrication and control of soft robots. Nature 521, 467–475. 10.1038/nature14543 26017446

[B29] Tang A. F. Li Y. Kong L. F. Cheng X. J. (2013). Vibration analysis of tendon-based parallel robot for processing. Adv. Mater. Res. 655, 1086–1091. 10.4028/www.scientific.net/amr.655-657.1086

[B30] Virginia Dignum K. P. Melanie P. Vosloo S. (2021). “Policy guidance on ai for children”

[B31] Wang Y. Wang Y. Mushtaq R. T. Wei Q. (2024). Advancements in soft robotics: a comprehensive review on actuation methods, materials, and applications. Polymers 16, 1087. 10.3390/polym16081087 38675005 PMC11054840

[B32] Webster R. J. Jones B. A. (2010). Design and kinematic modeling of constant curvature continuum robots: a review. Int. J. Robotics Res. 29, 1661–1683. 10.1177/0278364910368147

[B33] Xie S. Zhong H. Li Y. Xu S. (2024). An improved generalized prandtl–ishlinskii model for the hysteresis modeling of pneumatic artificial muscles. J. Intelligent Material Syst. Struct. 35, 1444–1455. 10.1177/1045389x241273011

[B34] Yavuz Ş. (2021). An improved vibration control method of a flexible non-uniform shaped manipulator. Simul. Model. Pract. Theory 111, 102348. 10.1016/j.simpat.2021.102348

